# Inhibiting eukaryotic ribosome biogenesis: Mining new tools for basic research and medical applications

**DOI:** 10.15698/mic2019.10.695

**Published:** 2019-08-20

**Authors:** Lisa Kofler, Michael Prattes, Helmut Bergler

**Affiliations:** 1 Institute of Molecular Biosciences, University of Graz, NAWI Graz, Humboldtstraβe 50-EG, A-8010 Graz, Austria.

**Keywords:** ribosome biogenesis, Inhibitors, yeast, high-throughput screen, rRNA processing

## Abstract

The formation of new ribosomes is a fundamental cellular process for each living cell and is tightly interwoven with cell cycle control and proliferation. Minimal disturbances of this pathway can result in ribosomopathies including an increased risk for certain cancer types. Thus, targeting ribosome biogenesis is an emerging strategy in cancer therapy. However, due to its complex nature, we are only at the beginning to understand the dynamics of the ribosome biogenesis pathway. One arising approach that will help us to embrace the tight timely cascade of events that is needed to form a new ribosome is the use of targeted chemical inhibition. However, only very few specific chemical inhibitors of the ribosome biogenesis pathway have been identified so far. Here we review our recently published screen to identify novel inhibitors of the ribosome biogenesis pathway in yeast (Awad *et al.*, 2019, BMC Biology). These inhibitors can provide novel tools for basic research and can serve as starting-points to develop new chemotherapeutics.

Ribosomes are ribonucleoprotein complexes in the MDa range and translate the genetic code into proteins. Hence, the efficient production of new ribosomes is essential for every growing and dividing cell. In eukaryotic cells, ribosome biogenesis requires around 250 non-ribosomal assembly factors and ranks among the most energy and resource demanding processes. Ribosome formation is linked with other fundamental circuits controlling cell cycle and proliferation through a tight regulatory network. Therefore, dysregulation of ribosome biogenesis in any direction (up- or downregulation) affects cell proliferation rate and can favor the unleashed growth of tumors or result in developmental disorders, anemia or bone marrow failure (in this context often called ribosomopathies). Fast proliferating cells, including cancer cells, strongly depend on high levels of ribosome biogenesis, whereas quiescent cells can presumably survive a temporary shutdown of the pathway with their available depot of mature ribosomes. Accordingly, the strong dependency on high levels of ribosome biogenesis represents an Achilles heel of cancer cells and its inhibition represents a promising strategy for the treatment of tumors or infections.

Eukaryotic ribosomes are composed of ~80 ribosomal proteins and four rRNAs that have to be thoroughly assembled in a sophisticated cellular manufacturing line. Nascent ribosomes start their maturation in the nucleolus, where the rRNAs are transcribed and rapidly loaded with the first assembly factors. The mature rRNAs are then carved out of the raw RNA precursor transcript in numerous processing steps. Cleavage at a specific site of the primary transcript gives birth to the precursors of the two ribosomal subunits, the pre-40S and the pre-60S particles, which are further assembled in two independent pathways. While the pre-40S precursor is rapidly transported into the cytoplasm, where the final maturation takes place, the pre-60S particle undergoes numerous rearrangements and rRNA processing steps until it is exported into the cytoplasm for finalization.

Despite the tremendous progress in our understanding of ribosome biogenesis in recent years, the full complexity and especially the dynamic nature of the maturation cascade still remain enigmatic and crave for novel approaches. Therefore, it is of great importance to understand the cascade of events during ribosome biogenesis and its regulatory network in detail. In the past, common genetic and biochemical studies, mostly done in yeast, gathered basic knowledge about ribosome formation including groundbreaking structural insights. However, the available tools provide rather static snapshots and hence are not suitable to unravel the dynamics of this rapid and interwoven pathway. One promising strategy to circumvent these limitations is the use of small molecular weight inhibitors that allow experimenters the rapid blockage of ribosome formation at a distinct step and to monitor the effects within minutes.

However, despite the large repertoire of potential targets provided by the ~250 maturation factors alone, only very few chemical inhibitors of ribosome biogenesis are known so far. With the help of diazaborine, the first inhibitor of eukaryotic ribosome biogenesis, our lab previously demonstrated the tremendous potential to investigate the dynamics of ribosome biogenesis by targeted chemical inhibition.

By inhibiting the AAA-ATPase Drg1 in the cytoplasm, diazaborine specifically blocks release and recycling of shuttling pre-60S maturation factors. Consequently, these shuttling factors are trapped on cytoplasmic particles, which leads to their depletion in the nucleus. The resulting shortage for the next round of ribosome biogenesis in the nucleus and can lead to secondary blocks, eventually causing entrapment of even earlier acting maturation factors. This rebound effect is therefore transmitted back step by step to the earliest stages of pre-ribosome formation. Since there is hardly any free pool of maturation factors, these secondary and tertiary blockages manifest within minutes. Unraveling this cascade of events was a crucial step to understand the coordination of nucleolar and cytoplasmic maturation events and demonstrated that the full dynamics of ribosome biogenesis can only be disentangled with the fast onset of chemical inhibition.

In order to find new tools for targeted chemical inhibition, we systematically screened collections of naturally occurring and/or clinically used substances for inhibitory effects on ribosome biogenesis in yeast using a microscopy-based *in vivo* screen (**Figure 1**). Our screening approach is based on the fact that perturbance of ribosome biogenesis often causes an entrapment of pre-ribosomal particles in the nucleoplasm or the nucleolus. Thus, to score for ribosomal particle maturation defects, we separately GFP-tagged selected ribosomal reporter proteins for the small and the large subunit. Since these ribosomal reporter proteins are components of mature ribosomes, the GFP-signal in untreated cells is located in the cytoplasm. Accordingly, a shift of the fluorescence signal to the nucleolus and/or nucleoplasm upon inhibitor treatment indicates a block in ribosome biogenesis. This simple basic setup allowed us to rapidly screen ~1000 compounds, an approach, which could be extended to larger compound collections.

**Figure 1:**
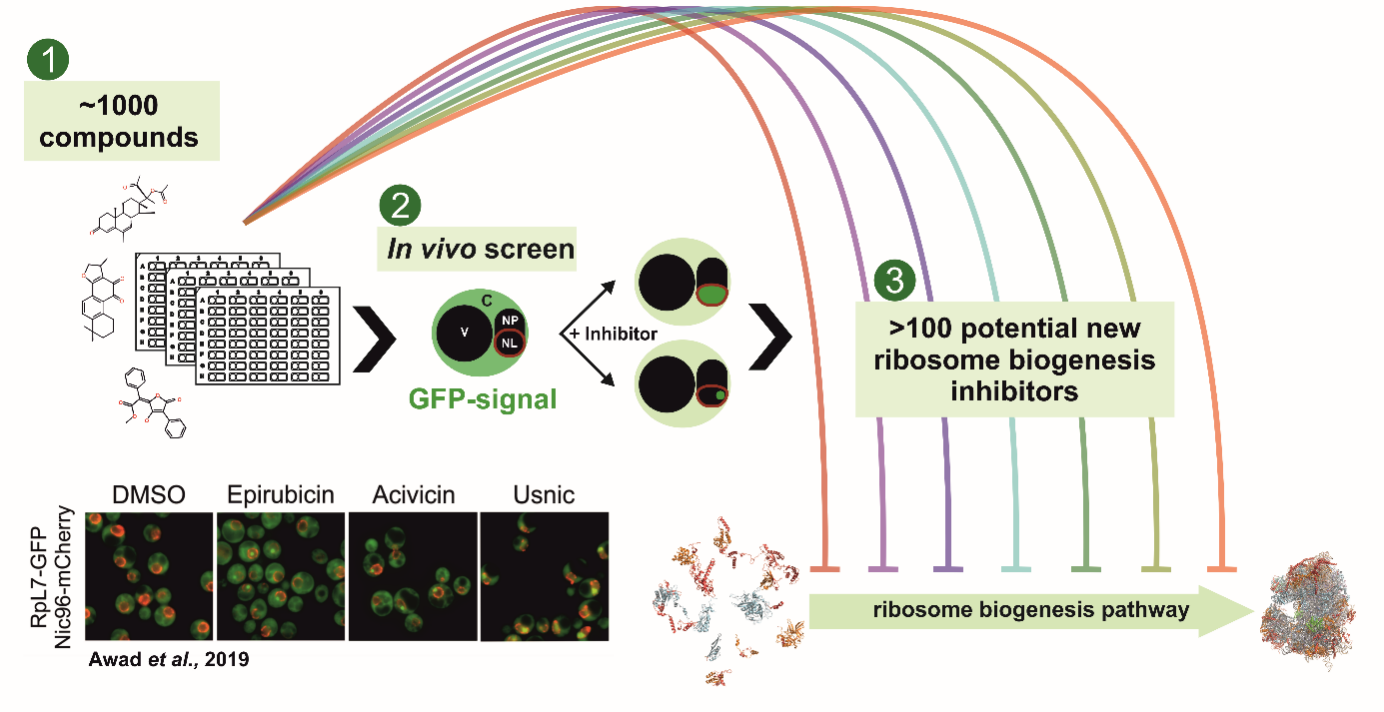
FIGURE 1: Microscopy-based screen to identify novel ribosome biogenesis inhibitors. ~1000 compounds of commercially available collections (Enzo and NIH) were tested for their ability to inhibit maturation of the large or small ribosomal subunits. Positively scoring compounds were indicated by a shift of the cytoplasmic (untreated) localization of GFP-tagged ribosomal reporter proteins into either the nucleolus or the nucleoplasm. The effects of the identified compounds were further characterized by northern blot analysis to detect specific rRNA processing defects, which gives a first hint at the inhibited maturation stage.

In the frame of this study, we identified 128 compounds inhibiting maturation of either the small or the large ribosomal subunit or both (indicative of a very early block in the pathway). Northern blot analysis demonstrated a broad spectrum of different rRNA processing defects caused by these inhibitors, indicating that the compounds affect a wide range of different maturation steps within the pathway. While some of the identified substances were already known to interfere with the RNA metabolism (e.g. Acivicin and Mycophenolic acid) or had been linked to ribosome biogenesis before (e.g. Vulpinic acid), the vast majority of compounds were for the first time recognized as inhibitors of the ribosome biogenesis pathway. Strikingly, many of the inhibitors identified in the screen were shown previously to affect tumor cell growth or had already been in clinical use for cancer treatment (e.g. Carmofur or Rubicin derivatives). This result suggests that ribosome biogenesis is an extremely promising pathway for antitumor chemotherapy and provides a rich source of novel inhibitor targets to be explored in future studies.

In sum, the work from Awad et al., 2019 delivers a first comprehensive set of ribosome biogenesis inhibitors to dissect the dynamic character of this pathway in basic research, but also establishes ribosome biogenesis as promising target for drug design.

